# Association of Non-Dipping Blood Pressure Patterns with Fetal Growth Restriction and Postpartum Chronic Hypertension in Gestational Hypertension

**DOI:** 10.3390/medicina62020414

**Published:** 2026-02-22

**Authors:** Ümeyir Savur, Ersin İbişoğlu, Haci Murat Güneş, Saime Güneş, Aykun Hakgor, Aysel Akhundova

**Affiliations:** 1 Department of Cardiology, Istanbul Medipol University, Medipol Mega University Hospital, Istanbul 34214, Turkey; drumeyirsavur@hotmail.com (Ü.S.); dr_muratgunes@hotmail.com (H.M.G.); aykunhakgor@gmail.com (A.H.); aysel.akhundova@yahoo.com (A.A.); 2 Department of Cardiology, Istanbul Health Sciences University, Başakşehir Cam and Sakura City Hospital, Istanbul 34480, Turkey; 3 Department of Radiology, Istanbul Health Sciences University, Başakşehir Cam and Sakura City Hospital, Istanbul 34480, Turkey; saimeturgut82@gmail.com

**Keywords:** ambulatory blood pressure monitoring, circadian blood pressure, fetal growth restriction, gestational hypertension, non-dipping blood pressure pattern, postpartum chronic hypertension, pregnancy hypertension

## Abstract

Background and Objectives: Gestational hypertension (GH) is increasingly recognized as an early manifestation of maternal cardiovascular vulnerability. Ambulatory blood pressure monitoring (ABPM) enables the evaluation of circadian blood pressure behavior, and a non-dipping blood pressure pattern (NDBP), defined as a nocturnal systolic decline of <10%, has been associated with endothelial dysfunction, placental hypoperfusion, and adverse pregnancy outcomes. However, the prognostic value of NDBP for postpartum chronic hypertension (PPCHT) remains insufficiently explored. Materials and Methods: This retrospective observational study included 196 women with gestational hypertension beyond 20 weeks of gestation who underwent ABPM between 2013 and 2025. Patients were classified as dippers (≥10% nocturnal systolic decline) or non-dippers (<10%). The primary outcome was postpartum chronic hypertension, defined as a persistent office blood pressure ≥ 140/90 mmHg or continued antihypertensive therapy at 12-month follow-ups. Secondary outcomes included fetal growth restriction (FGR), preeclampsia, and hypertensive complications. Univariable and multivariable logistic regression analyses were performed to identify independent predictors of PPCHT and FGR. Results: In the cohort, 124 women (63.3%) exhibited a non-dipping blood pressure pattern. At 12 months postpartum, 93 women (47.4%) developed chronic hypertension. Non-dipping was significantly more frequent among women with PPCHT compared with those that remained normotensive (75.3% vs. 52.4%). Non-dippers also demonstrated higher rates of fetal growth restriction and preeclampsia. In multivariable analysis, NDBP remained independently associated with PPCHT after adjustments for age and daytime blood pressure parameters. Furthermore, NDBP and elevated daytime systolic blood pressure were independent predictors of FGR. Conclusions: A non-dipping blood pressure pattern is highly prevalent in gestational hypertension and is independently associated with both fetal growth restriction and postpartum chronic hypertension. Incorporating ABPM-derived circadian blood pressure phenotyping into antenatal assessments may improve risk stratification and support targeted postpartum cardiovascular surveillance strategies.

## 1. Introduction

Gestational hypertension (GH), defined as a new-onset blood pressure elevation after 20 weeks of gestation in previously normotensive women, affects approximately 5–10% of all pregnancies and remains a leading contributor to maternal and fetal morbidity worldwide [1]. Although GH often resolves following childbirth, up to 40–50% of affected women subsequently develop postpartum chronic hypertension (PPCHT), which is associated with an increased long-term cardiovascular risk, including ischemic heart disease, heart failure, stroke, and premature mortality [2,3,4]. These observations have led to the growing recognition that hypertensive disorders of pregnancy represent an early manifestation of cardiovascular vulnerability, effectively serving as a clinical “stress test” of the maternal vascular system [5].

The circadian blood pressure (BP) rhythm provides a physiological dimension not captured by conventional office measurements. In healthy individuals, nocturnal BP typically falls by ≥10%, a profile termed the “dipper” pattern. When this decline is blunted (<10%), a non-dipping blood pressure pattern (NDBP) manifests, reflecting sympathetic overactivity, endothelial dysfunction, arterial stiffness, and low-grade vascular inflammation [6,7]. While NDBP is consistently associated with adverse cardiovascular outcomes and renal disease progression in non-pregnant populations [8], its role during pregnancy is increasingly linked to placental hypoperfusion, preeclampsia, and fetal growth restriction (FGR) [9,10].

Despite the established prognostic significance of GH, refined risk markers capable of identifying women at the highest risk for future cardiovascular complications remain incompletely defined. Previous research has predominantly focused on traditional clinical variables such as maternal age and hypertension severity [11]. In contrast, the prognostic relevance of nocturnal BP phenotypes, assessed via ambulatory blood pressure monitoring (ABPM), has not been sufficiently explored in relation to postpartum outcomes. Given its non-invasive nature and ability to capture circadian behavior, ABPM may offer critical complementary data for cardiovascular risk stratification.

Accordingly, this retrospective observational study aimed to (i) evaluate the association between ABPM-derived NDBP during pregnancy and the development of PPCHT at a 12-month follow-up and (ii) examine the relationship between NDBP and adverse pregnancy outcomes, specifically preeclampsia and fetal growth restriction.

## 2. Materials and Methods

### 2.1. Study Design

This retrospective observational study was conducted in accordance with the Strengthening the Reporting of Observational Studies in Epidemiology (STROBE) recommendations and was performed at a tertiary care, university-affiliated referral center with specialized obstetrics and cardiology outpatient clinics.

### 2.2. Study Population

**Figure 1:**
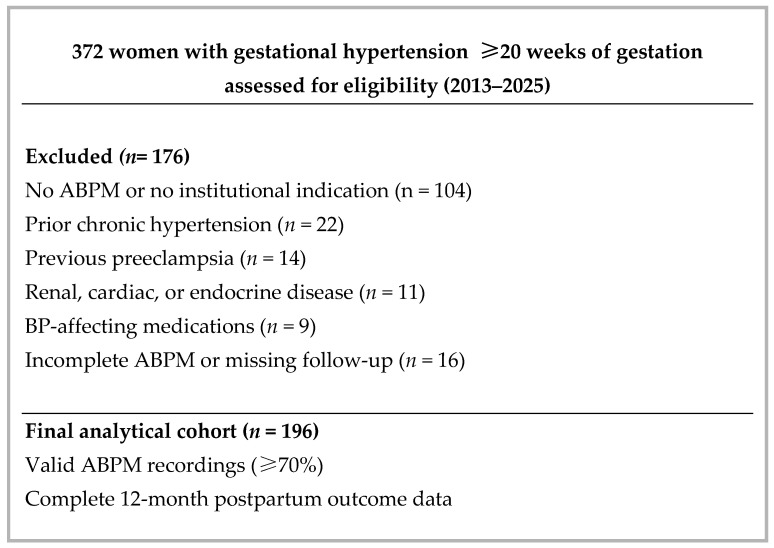
Flow diagram of patient selection.

Pregnant women followed in obstetrics and cardiology outpatient clinics between 2013 and 2025 were screened for eligibility according to predefined criteria. A total of 372 women beyond 20 weeks of gestation were screened. Of these, 196 women who underwent ambulatory blood pressure monitoring (ABPM) according to institutional clinical indications (e.g., suspected white-coat hypertension or evaluation of nocturnal blood pressure behavior) were included in the final analytical cohort. During the study period, ABPM was offered to all patients meeting these indications. Eligibility was limited to patients with complete and valid ABPM recordings (≥70% of scheduled measurements) and verifiable 12-month postpartum clinical outcome data within the institutional electronic health record system. The patient selection process is summarized in Figure 1.

Exclusion criteria included: (i) a prior diagnosis of chronic hypertension; (ii) a history of preeclampsia in a previous pregnancy; (iii) known renal, cardiac, or endocrine disorders; or (iv) the use of chronic pharmacological agents known to interfere with blood pressure regulation. Patients with incomplete baseline or follow-up data were also excluded.

### 2.3. ABPM Protocol

Ambulatory blood pressure monitoring was performed as part of routine clinical care for women with gestational hypertension, at the discretion of the treating obstetrician or cardiologist. Indications included: (1) confirmation of blood pressure control, (2) assessment of hypertension severity, and (3) evaluation of circadian blood pressure patterns in cases of suspected abnormal nocturnal behavior. To minimize potential selection bias, ABPM was offered to all eligible patients meeting these criteria during the study period. ABPM was performed using the Tono-port V system (General Electric Healthcare, Chicago, IL, USA). To ensure measurement accuracy, appropriately sized cuffs were placed on the participants’ non-dominant arm. The devices were programmed to record blood pressure at 30 min intervals during the daytime (06:00 to 23:00) and at 60 min intervals during the nighttime (23:00 to 06:00). ABPM recordings were considered valid only if at least 70% of the programmed measurements were successfully captured. This threshold was established to ensure a representative assessment of the 24 h blood pressure profile.

Hypertension on ABPM was defined according to the 2023 European Society of Hypertension (ESH) guideline thresholds: a 24 h mean BP ≥ 130/80 mmHg, a daytime mean ≥ 135/85 mmHg, or a nighttime mean ≥ 120/70 mmHg [12]. For each participant, systolic blood pressure (SBP), diastolic blood pressure (DBP), and mean arterial pressure (MAP) were calculated for the 24 h, daytime, and nighttime periods.

The nocturnal BP dipping rate was calculated as follows: (Mean Daytime SBP − Mean Nighttime SBP)/Mean Daytime SBP × 100. In accordance with standard physiological definitions, a nocturnal decline of <10% in mean systolic blood pressure was categorized as a non-dipping blood pressure pattern, whereas a decline of ≥10% was defined as a dipper pattern [13].

### 2.4. Outcome Definitions

The primary outcome was postpartum chronic hypertension, defined as the persistence of office blood pressure ≥ 140/90 mmHg or the continued requirement for antihypertensive therapy at the 12-month follow-up.

To ensure a standardized and reproducible assessment, office BP measurements were obtained during routine postpartum visits using a calibrated automated oscillometric device, in accordance with the 2024 European Society of Cardiology (ESC) Guidelines for the Management of Elevated Blood Pressure and Hypertension [14]. Participants were seated with back support and the arm positioned at heart level after at least 5 min of quiet rest prior to measurement. Three consecutive readings were recorded at 1–2 min intervals; the first reading was discarded, and the average of the final two readings was used for analysis. All measurements were performed by the same trained nursing staff to minimize interobserver variability. The diagnosis of PPCHT was subsequently confirmed and documented in the institutional electronic medical record by the treating physician. All documented cases were crossreferenced with antihypertensive medication prescriptions to ensure diagnostic accuracy.

Secondary outcomes were defined as follows:

- Preeclampsia: Diagnosed according to established clinical criteria including new-onset hypertension (≥140/90 mmHg) after 20 weeks of gestation combined with proteinuria or other systemic dysfunction as documented in the medical records [1].
- Fetal Growth Restriction: Defined as an ultrasonographic estimated fetal weight below the 10th percentile for gestational age, leading to a clinical diagnosis of impaired fetal growth [15].
- Hypertensive Complications: Included any unscheduled maternal hospitalization required for blood pressure stabilization or management of acute hypertensive episodes during pregnancy.

### 2.5. Statistical Analysis

Statistical analyses were performed using IBM SPSS Statistics (Version 26.0; IBM Corp., Armonk, NY, USA). The normality of data distribution was evaluated both using the Kolmogorov–Smirnov test and considering histogram curves. Continuous variables are presented as mean ± standard deviation for normally distributed data and as median (interquartile range [IQR]) for non-normally distributed data. Categorical variables are expressed as absolute numbers and percentages, with comparisons performed using the Pearson chi-square test or Fisher’s exact test, as appropriate. Between-group differences for continuous variables were assessed using the independent samples *t*-test or the Mann–Whitney U test, depending on the normality of the distribution. To identify independent predictors of PPCHT and FGR, univariable and multivariable binary logistic regression analyses were conducted. Clinically significant variables with a *p* Value < 0.05 in univariable analysis were included in the multivariable models to calculate adjusted odds ratios (ORs) and 95% confidence intervals (CIs). The presence of substantial collinearity among the independent variables included in the multivariate logistic regression models was investigated by calculating collinearity tolerance and variance inflation factors (VIFs) (Supplementary Table S1). Given the retrospective nature of this study, no a priori power analysis was conducted; however, the final sample size was considered sufficient for the planned regression models. Statistical significance was defined as a two-sided *p* Value < 0.05.

## 3. Results

### 3.1. Baseline Characteristics and Blood Pressure Patterns

A total of 196 women with gestational hypertension were included in this analysis. According to ambulatory blood pressure monitoring, 72 patients (36.7%) exhibited a dipper blood pressure pattern, while 124 patients (63.3%) demonstrated a non-dipper blood pressure pattern.

Patients with a non-dipping pattern had significantly higher rates of fetal growth restriction, preeclampsia, and postpartum chronic hypertension compared with those exhibiting a dipper pattern. In addition, all day and nighttime systolic, diastolic, and mean blood pressure values, as well as the prevalence of nocturnal hypertension, were significantly higher in the non-dipper group. The maternal age and gestational week at the time of ABPM did not differ significantly between groups.

Table 1 summarizes baseline characteristics, blood pressure parameters, and clinical outcomes according to the dipping status.

### 3.2. Comparison According to Postpartum Chronic Hypertension Status

At the 12-month postpartum follow-up, 93 women (47.4%) were diagnosed with postpartum chronic hypertension, while 103 women (52.6%) remained normotensive. Patients with PPCHT were older and exhibited significantly higher all day, daytime, and nighttime systolic, diastolic, and mean blood pressure values compared with those without PPCHT. The prevalence of a non-dipping blood pressure pattern was significantly higher among patients with PPCHT (75.3% vs. 52.4%).

Clinical characteristics and ambulatory blood pressure parameters according to the PPCHT status are presented in Table 2.

### 3.3. Univariable Predictors of Postpartum Chronic Hypertension

The univariable logistic regression analysis demonstrated that age, all day systolic and diastolic blood pressure, daytime and nighttime blood pressure parameters, and the presence of a non-dipping blood pressure pattern were significantly associated with the development of PPCHT.

Univariable associations with PPCHT are detailed in Table 3.

### 3.4. Multivariable Analysis of Blood Pressure Patterns and PPCHT

In the multivariable logistic regression analysis, the daytime systolic blood pressure, daytime diastolic blood pressure, age, and non-dipping blood pressure pattern remained independently associated with PPCHT.

Results of the multivariable analysis are shown in Table 4.

### 3.5. Association Between Blood Pressure Patterns and Fetal Growth Restriction

The univariable analysis revealed significant associations between fetal growth restriction and the daytime systolic blood pressure, daytime diastolic blood pressure, and non-dipping blood pressure pattern. In the multivariable analysis, the daytime systolic blood pressure and non-dipping pattern remained independently associated with FGR.

These associations are presented in Table 5.

## 4. Discussion

Gestational hypertension has increasingly been reclassified from an isolated obstetric condition to a sentinel marker of future cardiovascular disease, reflecting underlying endothelial injury, autonomic dysregulation, and vascular maladaptation. Large cohort studies have demonstrated that hypertensive disorders of pregnancy are associated with a long-term cardiovascular disease burden comparable in magnitude to traditional cardiometabolic risk factors [16]. In this context, the present study adds to existing evidence by demonstrating that an ambulatory blood pressure-defined non-dipping pattern is highly prevalent among women with GH and is strongly associated with adverse pregnancy outcomes as well as the development of PPCHT. In our cohort, nearly two-thirds of women with GH exhibited a non-dipping blood pressure pattern.

The strengths of this study include the use of ambulatory blood pressure monitoring to characterize circadian blood pressure patterns, the relatively long postpartum follow-up period, and the simultaneous assessment of both maternal cardiovascular and fetal outcomes in a well-defined gestational hypertension cohort.

Non-dipping blood pressure physiology has been consistently linked to adverse cardiovascular outcomes in non-pregnant adults, including increased arterial stiffness, left ventricular remodeling, and renal microvascular damage [17]. Sympathetic hyperactivity and the enhanced nocturnal activation of the renin–angiotensin–aldosterone system are thought to contribute to impaired nocturnal blood pressure reduction, thereby promoting sustained vascular stress [18]. These mechanisms are particularly relevant in pregnancy, a state characterized by profound hemodynamic and vascular adaptation, in which excessive endothelial activation, oxidative stress, and inflammatory pathways have been implicated in the pathogenesis of hypertensive disorders [19]. Although biomarkers of endothelial dysfunction or oxidative stress were not available in the present dataset, the strong association observed between NDBP and both maternal and fetal outcomes suggests that abnormal nocturnal blood pressure regulation may reflect a subclinical vascular dysfunction already present during gestation.

In this context, an altered redox balance and circulating plasma factors may represent potential mechanistic links between abnormal nocturnal blood pressure regulation and adverse maternal fetal outcomes. Recent evidence suggests that pregnancies complicated by hypertensive disorders are characterized by increased oxidative stress, endothelial activation, and the presence of harmful circulating factors capable of impairing vascular and placental function. Notably, Grossini et al. demonstrated that plasma from high-risk pregnancies exhibits an altered redox profile and exerts detrimental effects on umbilical cord-derived mesenchymal stem cells, supporting a role for circulating redox-mediated mechanisms in pregnancy-related vascular dysfunction [20].

The prognostic significance of nocturnal blood pressure abnormalities during pregnancy remains incompletely characterized. Nevertheless, emerging evidence indicates that altered nocturnal blood pressure patterns may be associated with impaired uteroplacental perfusion [21], dysregulated placental angiogenic balance—including elevated sFlt-1/PlGF ratios [22]—and an increased likelihood of hypertensive complications requiring hospitalization [23]. In line with these observations, our findings demonstrate a markedly higher incidence of FGR among women exhibiting a non-dipping blood pressure pattern. Importantly, NDBP remained a strong and independent predictor of FGR after adjustments for daytime blood pressure parameters, supporting a potential link between circadian blood pressure dysregulation and placental insufficiency.

Beyond pregnancy-related outcomes, the present study highlights the clinical relevance of NDBP for postpartum cardiovascular risk. Nearly half of the study population developed PPCHT at the 12-month follow-up, underscoring the substantial burden of persistent hypertension following GH. Women with PPCHT exhibited significantly higher ambulatory blood pressure values across all time periods and were significantly more likely to demonstrate a non-dipping pattern. Notably, NDBP remained independently associated with PPCHT even after the adjustment for age and daytime blood pressure parameters, suggesting that nocturnal blood pressure behavior provides prognostic information beyond conventional clinic or daytime measurements.

Postpartum hypertension remains substantially underdiagnosed in routine clinical practice. Recent studies using remote monitoring and telehealth-based strategies have shown that a considerable proportion of women meeting diagnostic criteria for PPCHT fail to attend scheduled follow-up visits within the first postpartum year [24]. In this context, ABPM-guided phenotyping during pregnancy may offer a practical and low-cost approach to identify women at a higher risk for persistent hypertension, enabling more targeted postpartum surveillance, lifestyle counseling, and cardiovascular risk modification strategies. Although blood pressure chronotherapy has demonstrated benefits in selected hypertensive populations [25], the present study was not designed to evaluate treatment timing or interventional strategies, and such approaches warrant investigation in future prospective trials.

Current antenatal risk stratification models for GH predominantly rely on office blood pressure measurements, proteinuria, and the occurrence of pregnancy-related complications, without incorporating circadian hemodynamic patterns. If confirmed in larger, prospective, multicenter cohorts, the integration of ABPM-derived nocturnal blood pressure phenotypes may represent a clinically meaningful addition to postpartum cardiovascular risk assessment. Furthermore, NDBP could potentially be incorporated into multifactorial risk models combining maternal characteristics, ambulatory blood pressure parameters, placental biomarkers, and indices of vascular function.

## 5. Conclusions

In women with gestational hypertension, a non-dipping blood pressure pattern identified by ambulatory blood pressure monitoring during pregnancy was common and independently associated with an increased risk of fetal growth restriction and postpartum chronic hypertension. These findings suggest that the assessment of circadian blood pressure patterns may provide clinically relevant information beyond conventional office measurements and help identify women who may benefit from a closer postpartum cardiovascular follow-up, pending confirmation in prospective studies.

## 6. Limitations

The primary limitations of this study include its retrospective, single-center design, which may introduce residual confounding and selection bias. Consequently, the sample size was determined by the availability of eligible patients with complete clinical and ABPM data rather than a predefined power calculation. Important demographic and biochemical variables, including the body mass index, smoking status, and baseline laboratory parameters, were not available and could not be incorporated into the multivariable analyses. In addition, ambulatory blood pressure monitoring was performed at a single time point during pregnancy, precluding a longitudinal assessment of circadian blood pressure patterns. Given the observational nature of this study, causality cannot be inferred, and the findings should be considered hypothesis-generating pending confirmation in prospective multicenter cohorts.

## Figures and Tables

**Table 1:** The characterization of the occurrence of the event and chi-square test results.

	DBP N: 72, (36.7%) Mean ± Sd	NDBP N: 124, (63.3%) Mean ± Sd	*p*
FGR	21 (29.2%)	83 (66.9%)	<0.001
Preeclampsia	15 (20.8%)	60 (48.4%)	<0.001
PPCHT	23 (31.9%)	70 (56.5%)	0.001
Age	31.17 ± 5.59	31.37 ± 5.61	0.806
GW	25.04 ± 2.79	24.38 ± 2.79	0.111
ADS	129.25 ± 11.86	135.81 ± 8.65	<0.001
ADD	86.29 ± 8.09	89.18 ± 7.45	0.012
DTS	134.60 ± 8.18	136.39 ± 8.81	0.161
DTD	89.28 ± 6.53	89.06 ± 7.27	0.832
DTM	104.38 ± 7.18	104.34 ± 6.83	0.971
NTS	113.54 ± 7.96	135.06 ± 9.33	<0.001
NTD	76 ± 7.95	86.24 ± 10.32	<0.001
NTM	90.14 ± 6.09	103.21 ± 7.88	<0.001
NHT	34 (47.2%)	113 (91.1%)	<0.001

ADD: all day diastolic blood pressure, ADS: all day systolic blood pressure, DTD: daytime diastolic blood pressure, DTM: daytime mean blood pressure, DTS: daytime systolic blood pressure, GW: gestational week, FGR: fetal growth restriction, NDBP: non-dipper blood pressure, NHT: nocturnal hypertension, NTD: nighttime diastolic blood pressure, NTM: nighttime mean blood pressure, NTS: nighttime systolic blood pressure, and PPCHT: postpartum chronic hypertension.

**Table 2:** Features of patients with and without PPCHT.

	PPCHT (+) N: 93, (47.4%)	PPCHT (−) N: 103, (52.6%)	*p*
Age	32.99 ± 5.11	29.77 ± 5.60	<0.001
GW	24.73 ± 2.92	24.52 ± 2.69	0.607
ADS	136.56 ± 8.97	130.54 ± 10.84	<0.001
ADD	90.97 ± 7.38	85.54 ± 7.28	<0.001
DTS	139.74 ± 7.94	132.11 ± 7.52	<0.001
DTD	91.86 ± 5.78	86.68 ± 7.11	<0.001
DTM	106.56 ± 6.69	102.36 ± 6.58	<0.001
NTS	129.48 ± 12.14	125.05 ± 14.61	0.023
NTD	84.81 ± 10.39	80.38 ±10.61	0.004
NTM	100.88 ± 9.84	96.17 ± 8.88	0.001
NDBP (+), n (%)	70 (75.3%)	54 (52.4%)	0.001

ADD: all day diastolic blood pressure, ADS: all day systolic blood pressure, DBP: dipper blood pressure, DTD: daytime diastolic blood pressure, DTM: daytime mean blood pressure, DTS: daytime systolic blood pressure, NDBP: non-dipper blood pressure, NTD: nighttime diastolic blood pressure, NTM: nighttime mean blood pressure, NTS: nighttime systolic blood pressure, and PPCHT: postpartum chronic hypertension.

**Table 3:** Binary logistic regression of PPCHT.

	OR (95% CI)	Univariate *p* Value
Age	1.118 (1.057–1.182)	<0.001
GW	1.027 (0.929–1.135)	0.605
ADS	1.063 (1.031–1.096)	<0.001
ADD	1.103 (1.059–1.150)	<0.001
NTS	1.025 (1.003–1.047)	0.024
NTD	1.041 (1.013–1.070)	0.004
NTM	1.055 (1.022–1.088)	0.001
NDBP	2.762 (1.501–5.080)	0.001
DTS	1.130 (1.084–1.178)	<0.001
DTD	1.152 (1.086–1.222)	<0.001

OR: odds ratio, CI: confidence interval, ADD: all day diastolic blood pressure, ADS: all day systolic blood pressure, DTD: daytime diastolic blood pressure, DTS: daytime systolic blood pressure, GW: gestational week, NTD: nighttime diastolic blood pressure, NTM: nighttime mean blood pressure, NTS: nighttime systolic blood pressure, NDBP: non-dipper blood pressure, and PPCHT: postpartum chronic hypertension.

**Table 4:** Association of PPCHT and blood pressure patterns.

	Univariate OR (95% CI)	*p* Value	Multivariate OR (95% CI)	*p* Value
DTS	1.130 (1.084–1.178)	<0.001	1.095 (1.041–1.153)	<0.001
DTD	1.152 (1.086–1.222)	<0.001	1.080 (1.011–1.153)	0.023
NDBP	2.762 (1.501–5.080)	0.001	3.128 (1.536–6.592)	0.002
Age	1.118 (1.057–1.182)	<0.001	1.128 (1.057–1.203)	<0.001

OR: odds ratio, CI: confidence interval, DTD: daytime diastolic blood pressure, DTS: daytime systolic blood pressure, NDBP: non-dipper blood pressure, and PPCHT: postpartum chronic hypertension.

**Table 5:** Association of FGR and blood pressure patterns.

	Univariate OR (95% CI)	*p* Value	Multivariate OR (95% CI)	*p* Value
DTS	1.109 (1.066–1.153)	<0.001	1.107 (1.054–1.163)	<0.001
DTD	1.071 (1.023–1.121)	0.003	1.010 (0.957–1.066)	0.716
NDBP	4.916 (2.616–9.241)	<0.001	5.388 (2.685–10.813)	<0.001

OR: odds ratio, CI: confidence interval, DTD: daytime diastolic blood pressure, DTS: daytime systolic blood pressure, FGR: fetal growth restriction, and NDBP: non-dipper blood pressure.

## Data Availability

The results of the datasets analyzed or created during this study are given in this article.

## Supplementary Materials

The following supporting information can be downloaded at https://www.mdpi.com/article/10.3390/medicina62020414/s1: Supplementary Table S1. Multicollinearity diagnostics for the multivariable logistic regression models.

## Abbreviations

The following abbreviations are used in this manuscript:

GH	Gestational Hypertension
PPCHT	Postpartum Chronic Hypertension
FGR	Fetal Growth Restriction
ABPM	Ambulatory Blood Pressure Monitoring
BP	Blood Pressure
NDBP	Non-Dipping Blood Pressure Pattern
SBP	Systolic Blood Pressure
DBP	Diastolic Blood Pressure
MAP	Mean Arterial Pressure
