# Supplementary material for: Association of Non-Dipping Blood Pressure Patterns with Fetal Growth Restriction and Postpartum Chronic Hypertension in Gestational Hypertension
Source: Medicina (Kaunas). 2026 Feb 22;62(2):414. doi: 10.3390/medicina62020414 (PMC12943090; doi:10.3390/medicina62020414)
Supplement: Supplementary file 1 [file medicina-62-00414-s001.zip › medicina-4151561-supplementary.pdf]

Supplementary Table S1. Multicollinearity diagnostics for the multivariable logistic regression models.

Model	Variable	Collinearity Tolerance	Variance Inflation Factor (VIF)
Model for PPCHT (Table 4)	DTS	0.664	1.505
	DTD	0.671	1.489
	Non-dipper	0.982	1.018
	Age	0.985	1.015
Model for FGR (Table 5)	DTS	0.667	1.500
	DTD	0.673	1.485
	Non-dipper	0.982	1.018

DTD: day time diastolic blood pressure, DTS: daytime systolic blood pressure, NDBP: Non-dipper blood pressure, PPCHT: postpartum chronic hypertension, FGR: fetal growth restriction.
